# Myo1g is required for efficient adhesion and migration of activated B lymphocytes to inguinal lymph nodes

**DOI:** 10.1038/s41598-021-85477-y

**Published:** 2021-03-30

**Authors:** D. Cruz-Zárate, O. López-Ortega, D. A. Girón-Pérez, A. M. Gonzalez-Suarez, J. L. García-Cordero, M. Schnoor, L. Santos-Argumedo

**Affiliations:** 1 Departamento de Biomedicina Molecular, Centro de Investigación y de Estudios Avanzados del Instituto Politécnico Nacional (CINVESTAV-IPN), Av. Instituto Politécnico Nacional 2508, San Pedro Zacatenco, 07360 Mexico City, Mexico; 2 Unidad Monterrey, Centro de Investigación y de Estudios Avanzados del Instituto Politécnico Nacional (CINVESTAV-IPN), Monterrey, NL Mexico; 3 Departmento and Posgrado en Inmunologia, Escuela Nacional de Ciencias Biologicas del Instituto Politécnico Nacional (ENCB-IPN), Mexico City, Mexico

**Keywords:** Cell biology, Immunology

## Abstract

Cell migration is a dynamic process that involves adhesion molecules and the deformation of the moving cell that depends on cytoskeletal remodeling and actin-modulating proteins such as myosins. In this work, we analyzed the role of the class I Myosin-1 g (Myo1g) in migratory processes of LPS + IL-4 activated B lymphocytes in vivo and in vitro. In vivo, the absence of Myo1g reduced homing of activated B lymphocytes into the inguinal lymph node. Using microchannel chambers and morphology analysis, we found that the lack of Myo1g caused adhesion and chemotaxis defects. Additionally, deficiency in Myo1g causes flaws in adopting a migratory morphology. Our results highlight the importance of Myo1g during B cell migration**.**

## Introduction

B lymphocytes circulate in the blood, lymph, and secondary lymphoid organs, including the spleen and lymph nodes^1, 2^. B lymphocytes bind to and then transmigrate through specialized blood venules known as high endothelial venules (HEV) to enter lymph nodes (LNs)^2^. Transmigration is a sequential process driven by the interaction between circulating B lymphocytes and HEV through several molecules, including chemokine receptors, selectins, and integrins^2–4^. Selectins regulate the capture and rolling of B lymphocytes on the endothelium, followed by firm adhesion mediated by integrins, and subsequent diapedesis across the wall of the HEV^3^. Different chemokines guide B lymphocytes and provide specificity during homing to LNs^4^. Chemokines are a family of proteins secreted by several cells that recruit immune cells to specific tissues^3, 5^.

CXCL12, CXCL13, and CCL19/21 are cytokines that drive the homing of B lymphocytes. These chemokines are recognized by their specific receptors CXCR4, CXCR5, and CCR7, in the B lymphocyte membrane. It has been reported that chemokine receptors' expression depends on the state of maturation of the B lymphocyte and homing stage^6, 7^. It has been suggested that each one plays a role within the B lymphocyte migration process^8, 9^. During migration, B lymphocytes change their morphology, which depends on the plasticity of the cell^10–12^. Among the factors that regulate cell plasticity, the interaction between the cytoskeleton and the plasma membrane is essential. Among the proteins regulating this association, myosins play an outstanding role^12, 13^. The myosin family includes class I and class II myosins^14, 15^. Class I myosins are proteins that can bind to both actin filaments and plasma membrane, thus regulating cellular functions that involve their interaction such as cell motility, cell shape control, sensory transduction, and vesicular trafficking^13, 16^. Myo1g is a class I myosin that is expressed in several immune cells, including resting and activated B lymphocytes^17^. Myo1g is known to regulate endocytosis and exocytosis of specific molecules; and adhesion to different substrates^18–21^. Myo1g-deficient activated B lymphocytes have decreased membrane tension and reduced elasticity^17^. Thus, in this work, we analyzed the participation of Myo1g during chemotaxis and transmigration, both in vivo and in vitro. Our results demonstrate that Myo1g deficiency significantly reduces the transmigration of LPS + IL-4 activated B lymphocytes into inguinal LNs. In vitro, Myo1g is essential for regulating surface expression of adhesion molecules and elasticity of activated B lymphocytes, allowing the correct formation of membrane projections and acquiring a migratory phenotype.

## Results

### The absence of Myo1g in activated B lymphocytes reduces their ability to interact with the HEV endothelium

**Figure 1:**
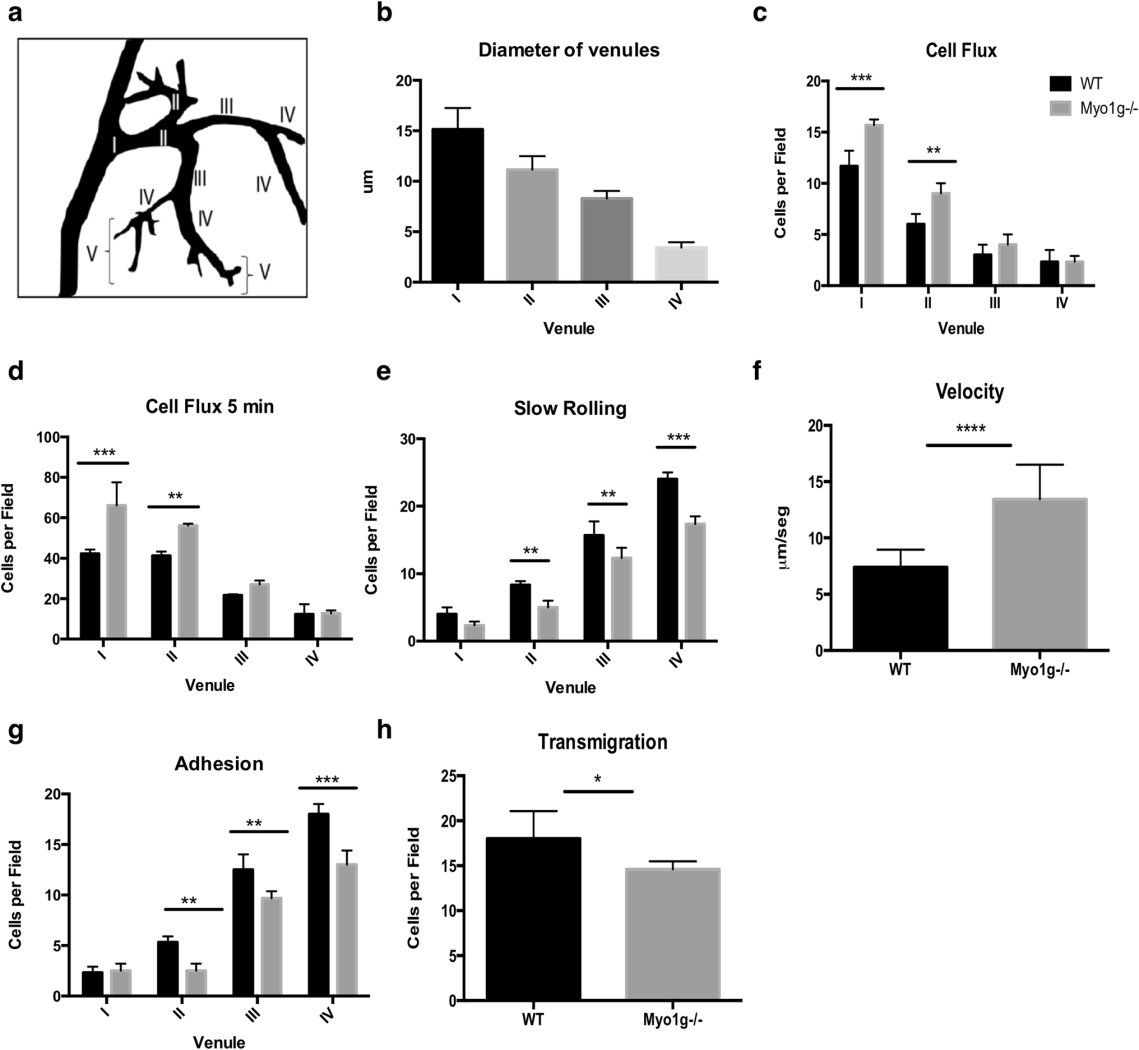
The absence of Myo1g affects adhesion and rolling of activated B lymphocytes on high endothelial venules. (**a**) Representative diagram of the different HEVs and (**b**) measurement of the diameter of venules (from the narrow IV to wide I) in the WT host mice's inguinal lymph nodes. LPS + IL-4 activated B cells (stained with Hoechst 33342) from WT or Myo1g^−/−^ mice were directly injected into the carotid artery of a WT mouse. One hour previously, the inguinal lymph node was injected with 100 μl CXCL13 (25 ng/ml). Measurements of B cell flux (frequency of leukocytes that pass through the postcapillary venules) for 1 min (**c**) and 5 min (**d**). (**e**) Quantification of the number of activated B cells from WT and Myo1g^−/−^ mice, performing slow rolling (frequency of leukocytes with a rolling velocity less than 5 μm/s) in the different venules (IV to I) of an inguinal lymph node of a host WT mouse. (**f**) Measurements of rolling velocities (traveling time from venules IV to I) of activated B cells from WT and Myo1g^-/-^ mice in the WT inguinal lymph node. (**g**) Quantification of adherent B cells in the different postcapillary venules after 45 min. (**h**) Quantification of extravasated B cells at 45 min. n = 3. Data are presented as mean ± s.d. *P < 0.05, **P < 0.01, ***P < 0.001.

Class I myosins regulate various cellular functions^16^, such as endocytosis and exocytosis of specific molecules, mobilization of recycling vesicles, and membrane tension of lymphocytes^17, 18, 21, 22^. Although Myo1g has been studied in several B lymphocytes' functions, its participation in the early stages of transmigration to secondary lymphoid organs has not been evaluated. In previous work, we observed that LPS + IL-4 activated Myo1g^−/−^ B lymphocytes had reduced migration in vitro and lower polarization of CXCR5 in the membrane^17, 21^. In mature B lymphocytes, the expression of CXCR5 and its function is conserved during the B lymphocyte homing to LNs^2, 23^. Thus, we analyzed whether Myo1g regulates LPS + IL-4 activated B lymphocyte motility on the HEV by IVM of CXCL13-stimulated inguinal LNs. In Fig. 1a,b, we show a schematic representation of HEV venules and their diameter. Activated Myo1g^−/−^ B lymphocytes showed increased cell flux only through venules I and II compared to WT (Fig. 1c,d). Of note, fewer activated B lymphocytes performed slow rolling in the absence of Myo1g with significant changes in venules II–IV (Fig. 1e). Those rolling Myo1g^−/−^ B lymphocytes in venules II–IV showed significantly higher rolling velocities than WT B lymphocytes (Fig. 1f). Higher rolling velocities in these venules translated into less firm adhesion (Fig. 1g) and reduced numbers of activated Myo1g^−/−^ B lymphocytes that transmigrated into inguinal LNs (Fig. 1h). This result agrees with what we previously reported that in the absence of Myo1g^−/−^ LPS + IL-4 activated B lymphocytes reached lymph nodes less efficiently^20, 21^. Besides, in an in vitro model of CXCL12-dependent migration, activated Myo1g^−/−^ B lymphocytes had a lower percentage of transmigration (Supplementary Figure S1), suggesting that the same migration defect in vivo could be present with other chemokines. Representative videos can be found as Supplementary Videos S2, S3.

### B lymphocytes require Myo1g for the expression of specific adhesion molecules upon activation

**Figure 2:**
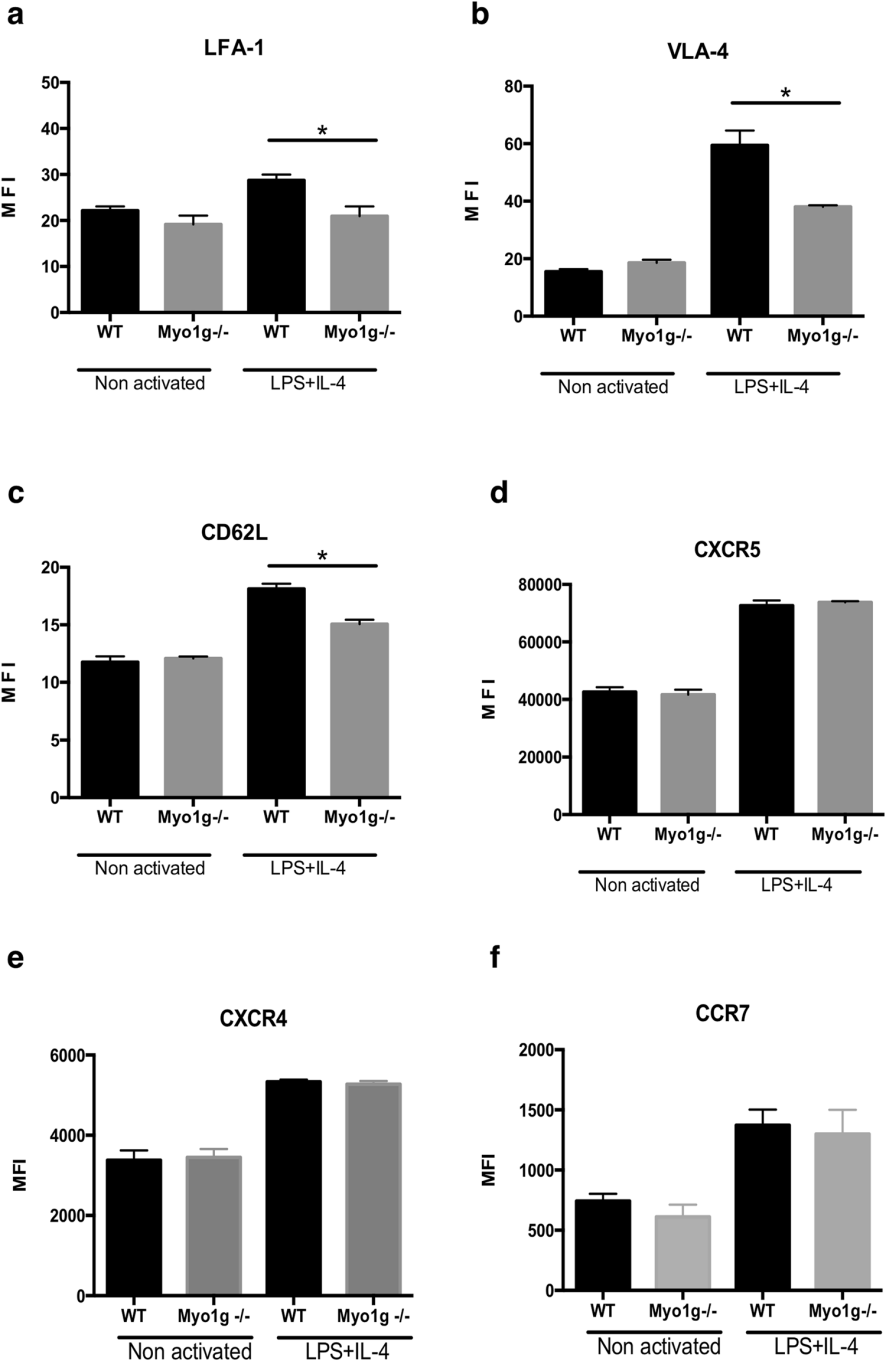
Myo1g is necessary for the surface expression of adhesion molecules in activated B lymphocytes. Graphical representation of mean fluorescence intensity values of (**a**) LFA-1, (**b**) VLA-4, (**c**) CD62L, (**d**) CXCR5, (**e**) CXCR4, (**f**) CCR7 in resting and LPS + IL-4 activated WT and Myo1g^−/−^ B lymphocytes (n = 4). Data are presented as mean ± s.d. *P < 0.05.

Due to the previous result and because the adhesion of B lymphocytes to the endothelium depends on the adhesion molecules, we decided to evaluate the surface expression of CD62L, LFA-1, and VLA-4 on WT and Myo1g^−/−^ B lymphocytes. Under basal conditions, we did not observe differences in the expression of these molecules. However, when we activated B cells with LPS and IL-4, Myo1g^−/−^ B lymphocytes showed reduced expression of CD62L, LFA-1, and VLA-4 in comparison with WT B lymphocytes (Fig. 2a–c). These results could explain the reduced adhesive interactions with the endothelium in HEV II, III, and IV observed in vivo (Fig. 1). Interestingly, we did not observe differences in the expression of CXCR5, CXCR4 and CCR7 (Fig. 2d–f) between resting and LPS + IL-4 activated WT and Myo1g^−/−^ B lymphocytes, suggesting that Myo1g^−/−^ B cells are in principle able to respond to CXCL13 gradient as well as other chemokines. Representative histograms can be found as Supplementary Figure S4.

### The absence of Myo1g alters the 3D migratory behavior of activated B lymphocytes

**Figure 3:**
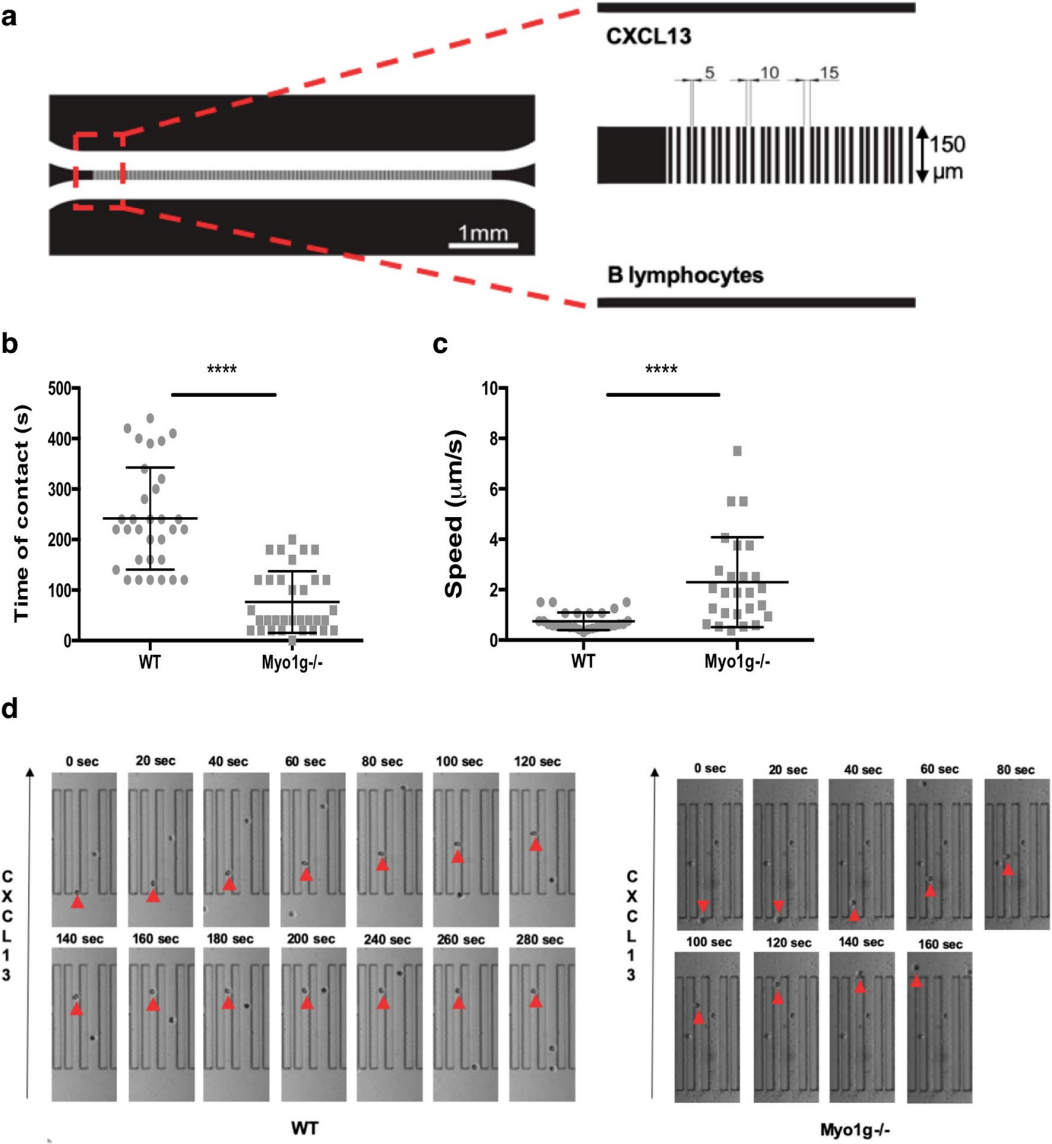
In the absence of Myo1g, activated B lymphocytes have altered motility in a confined 3D microchannel. (**a**) A general model of the microfluidic chamber. This device has 96 channels with gap widths of 5, 10, and 15 μm. Each channel is 150 μm long. Briefly, LPS + IL-4 activated WT or Myo1g^−/−^ B lymphocytes are placed by gravity inside the chamber. Then, CXCL13 is applied on the opposite side. After that, migration is recorded by time-lapse microscopy. (**b**) Time of contact (s) of activated WT or Myo1g^−/−^ B lymphocytes with the 10 μm microchannel wall. (**c**) speed (μm/s) of activated B lymphocytes moving through a 10 μm microchannel calculated while the cells were moving within the microchannel. (**d**) Representative still images showing displacement of the cells over time; (red arrows). Data are presented as mean ± s.d. ****P < 0.0001.

For the evaluation of cell motility, there are 2D and 3D migration models. In both models, cells' intrinsic factors participate, such as cell contractility, adhesion, and cytoskeleton rearrangements. Both models resemble conditions in vivo. However, 2D migration involves a process dependent on integrins and protrusion-adhesion-contraction. In contrast, in the case of 3D migration, a treadmilling effect has been seen where the actin flow is continuous^24, 25^. To analyze the motility of B lymphocytes in a confined microchannel, we used a custom-made microfluidic chamber. These devices, coated with ICAM-1 or Fibronectin, have been used to imaging lymphocytes “walking” through brief contacts with the microchannel walls during migration^26^. Microchannels connected two chambers with different widths (5, 10, and 15 μm), and CXCL13 was used as a chemoattractant to promote migration of cells from one chamber to the other (Fig. 3a). We observed that displacement could be better observed in 10 μm wide channels, suggesting that 15 μm channels were too broad and 5 μm channels too narrow for cell displacement. When analyzing LPS + IL-4 activated B lymphocytes that traveled through 10 μm microchannels, we observed that activated B lymphocytes "walk" interacting with the Fibronectin-coated microchannel walls, as reported for similar devices^26, 27^. When we analyzed activated Myo1g^−/−^ B lymphocytes, we observed reduced contact with the microchannel walls, affecting their speed (Fig. 3b,c). Figure 3d shows a series of representative time-lapse photographs of activated Myo1g^−/−^ and WT B lymphocytes displacement. In summary, these experiments indicated that Myo1g might function in controlling the interaction of activated B lymphocytes to the substrate for confinement-optimized motility. We observed similar behavior with CXCL12 as a chemoattractant (Supplementary Figure S5). Previous work has demonstrated that in the absence of Myo1g, T cells adhere less efficiently to ICAM-1 and migrate faster than WT T cells^27^, suggesting that the absence of Myo1g reduces the adhesive abilities of T and B lymphocytes. Similarly, the absence of Myo1g decreases the adhesion of B lymphocytes to other substrates^20, 21^. As a whole, these results could explain why we observed reduced adherence to HEV as well as lower transmigration of Myo1g^−/−^ B lymphocytes shown in Fig. 1. Representative videos can be found as Supplementary Videos S6, S7.

### Actin cytoskeletal remodeling upon activation depends on Myo1g

**Figure 4:**
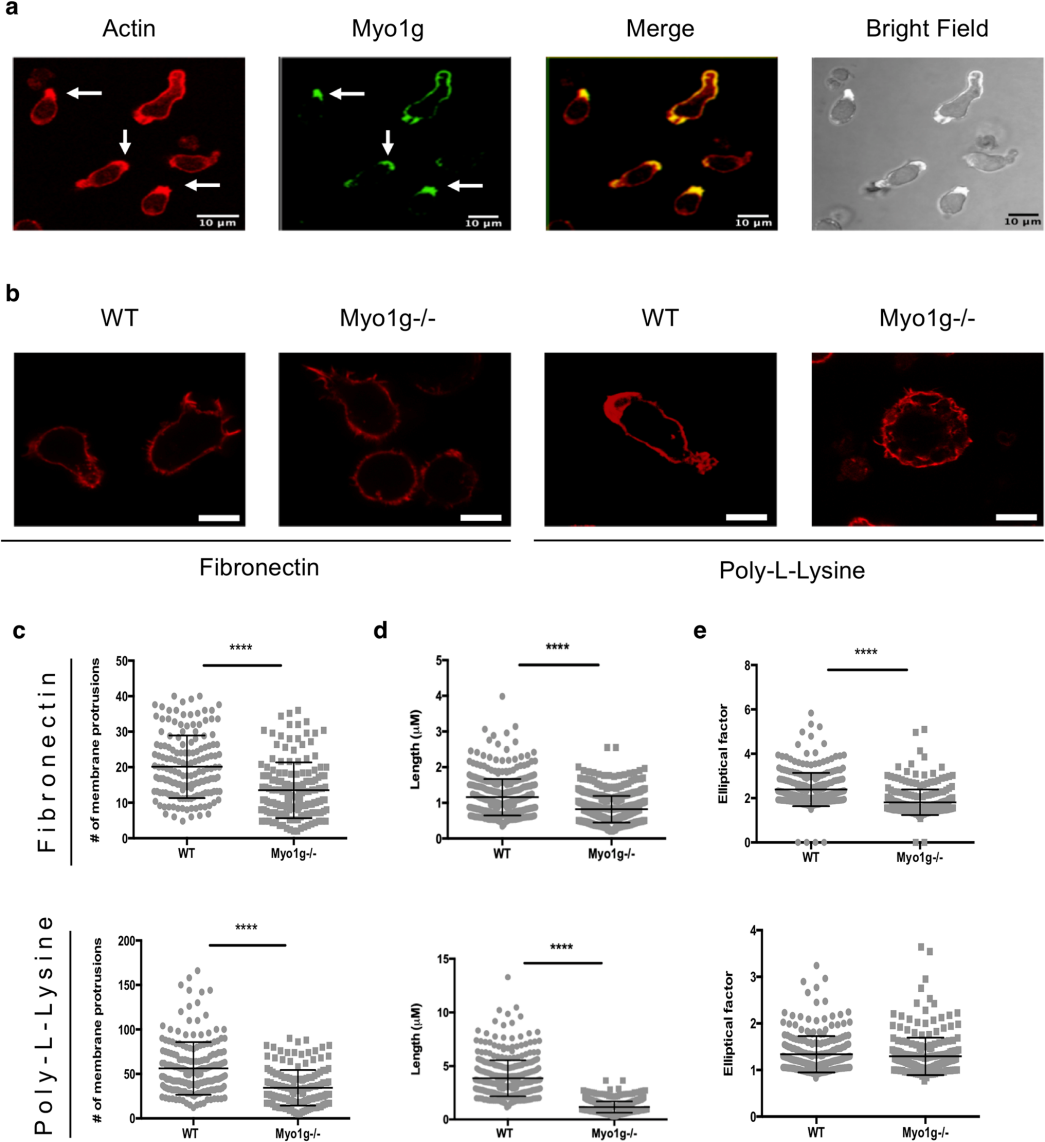
The absence of Myo1g affects the migratory phenotype of activated B lymphocytes. (**a**) Representative images (40 × objective) of migrating LPS + IL-4 activated WT B cells in a CXCL13 gradient. Myo1g is polarized toward the leading edge (white arrows). (**b**) Representative images (63 × objective) of activated WT or Myo1g^−/−^ B lymphocytes migrating on Fibronectin or Poly-l-Lysine coated slides in a CXCL13 gradient 30 min, followed by staining with TRITC-Phalloidin. Scale bars 5 μm. n = 3. (**c**) Measurement of the number of membrane protrusions, (**d**) length of protrusions, and (**e**) elliptical factor of CXCL13-stimulated WT or Myo1g^−/−^ B lymphocytes migrating on Fibronectin and Poly-l-Lysine coated slides. Data correspond to three independent experiments. Data are presented as mean ± s.d. ****P < 0.0001.

Lamellipodia and filopodia favor the initial interaction between B cells and the endothelium. Furthermore, the B cells must adopt a migratory phenotype and perform spreading on the endothelial cells to increase the contact area before starting to migrate^28^. Besides, B lymphocytes must modify their morphology during chemotactic migration, allowing amoeboid-like movements. Myo1g is known to regulate actin cytoskeletal dynamics and the plasma membrane's elasticity, critically involved in cytoskeletal changes^11, 20, 29, 30^. Therefore, we analyzed the distribution of Myo1g under conditions that favor a migratory phenotype. We incubated LPS + IL-4 activated B lymphocytes under a CXCL13 chemotactic gradient using a Zigmond chamber and analyzed migration for 30 min. Figure 4a shows how Myo1g accumulates at the leading edge of migrating activated B cells. Next, we evaluated whether the absence of Myo1g affected the B lymphocyte migratory phenotype. Representative photographs in Fig. 4b show the morphology of activated WT and Myo1g^−/−^ B lymphocytes on Fibronectin or Poly-l-Lysine under the CXCL13 chemotactic gradient. Moreover, when we analyzed the number and length of membrane projections in WT and Myo1g^−/−^ B lymphocytes, we observed a lower number and length of membrane projections in activated Myo1g^−/−^ B lymphocytes on both Fibronectin and Poly-l-Lysine substrates (Fig. 4c,d). To measure the cell's curvature, we used the length/width ratio (elliptic factor). An elliptic factor > 2 indicates an elongated morphology, which is characteristic of a moving lymphoid cell. On Fibronectin-coated slides, activated Myo1g^−/−^ B lymphocytes had a lower elliptical factor compared with activated WT B lymphocytes. Of note, no difference was observed on Poly-l-Lysine (Fig. 4e). These results suggest that in the absence of Myo1g, activated B lymphocytes have a reduced ability to modify their cytoskeleton and adopt a migratory phenotype. The differences observed in the assays described above when Fibronectin or Poly-l-Lysine coated slides were used also suggest the participation of integrins.

### Lack of Myo1g affects the ability of B lymphocytes to form membrane projections

**Figure 5:**
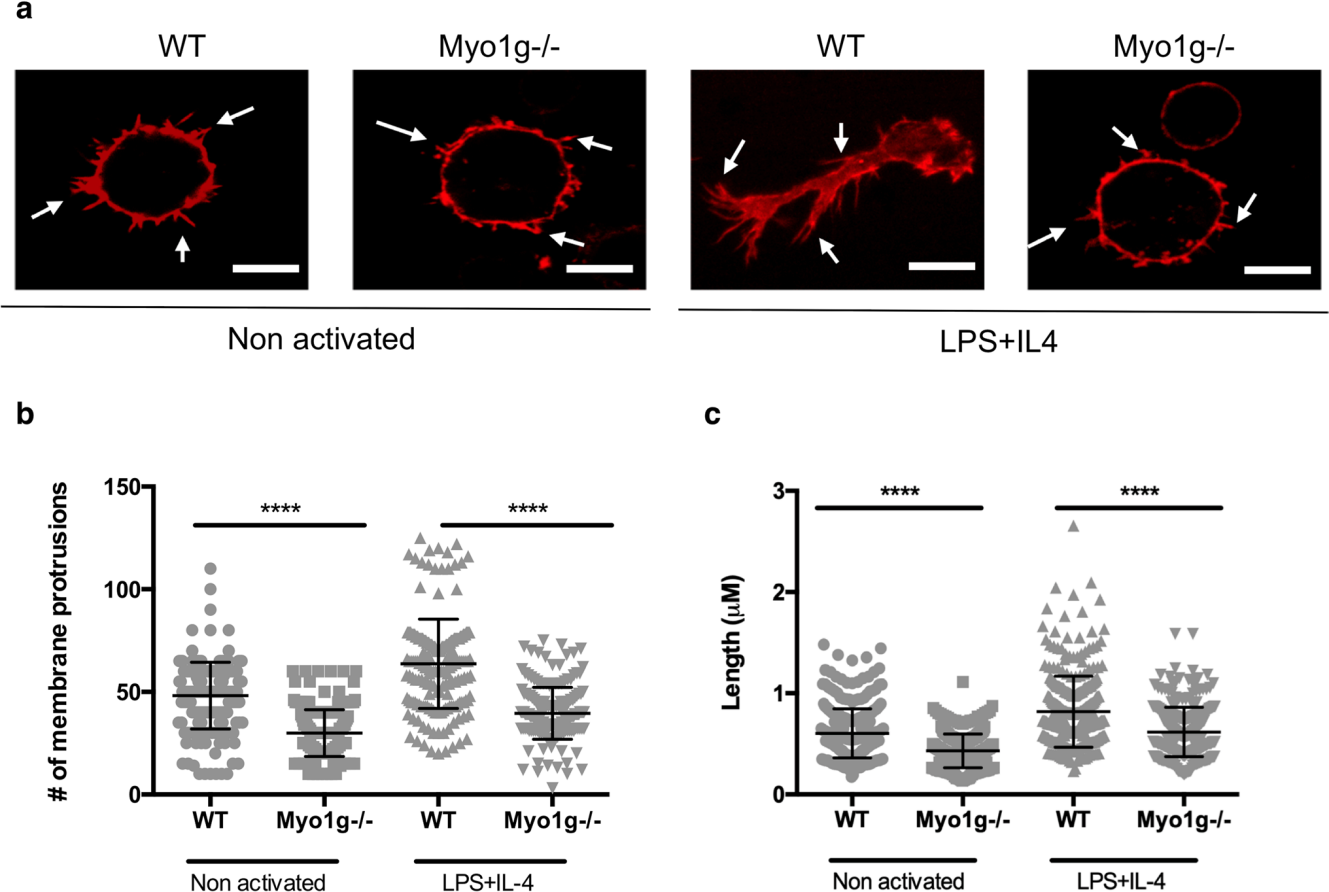
Myo1g participates in the ability of B lymphocytes to generate membrane protrusions. (**a**) Representative images (63 × objective) of non-activated or LPS + IL-4 activated WT and Myo1g^−/−^ B lymphocytes incubated two hours on Fibronectin-covered coverslips. Cells were stained with Tritc-Phalloidin, and membrane protrusions are indicated with white arrows. Scale bars 5 μm. n = 3. (**b**) The number of membrane protrusions and (**c**) their lengths were measured on at least 50 activated B lymphocytes of WT and Myo1g^−/−^ mice in three independent experiments. They were analyzed using ImageJ software (National Institutes of Health, Bethesda, MD). Data correspond to three independent experiments. Data are presented as mean ± s.d. ****P < 0.0001.

Because Myo1g has been shown to regulate membrane tension and thus form membrane projections^31^, we decided to evaluate the capacity to generate membrane projection in non-activated and activated B lymphocytes. We activated B cells on coverslips coated with Fibronectin. Figure 5a shows that both resting and LPS + IL-4 activated Myo1g^−/−^ and WT B lymphocytes can generate filopodia-like structures. However, the number of membrane projections and their lengths were significantly lower in Myo1g^−/−^ B lymphocytes in both conditions (Fig. 5b,c). Besides, evaluating curvature of B lymphocytes in both conditions using the elliptical factor, Myo1g^−/−^ B lymphocytes had a lower index, suggesting rounder shape (Supplementary Figure S8). These results suggest that B lymphocytes have defects in cytoskeletal dynamics in the absence of Myo1g. As we also observed that Myo1g participates in regulating the surface expression of adhesion molecules such as CD62L, VLA-4, and LFA-1 (Fig. 2a–c), these data may explain the reduced capacity of LPS + IL-4 activated Myo1g-deficient B lymphocytes to interact with the endothelium of HEV^20^.

## Discussion

**Figure 6:**
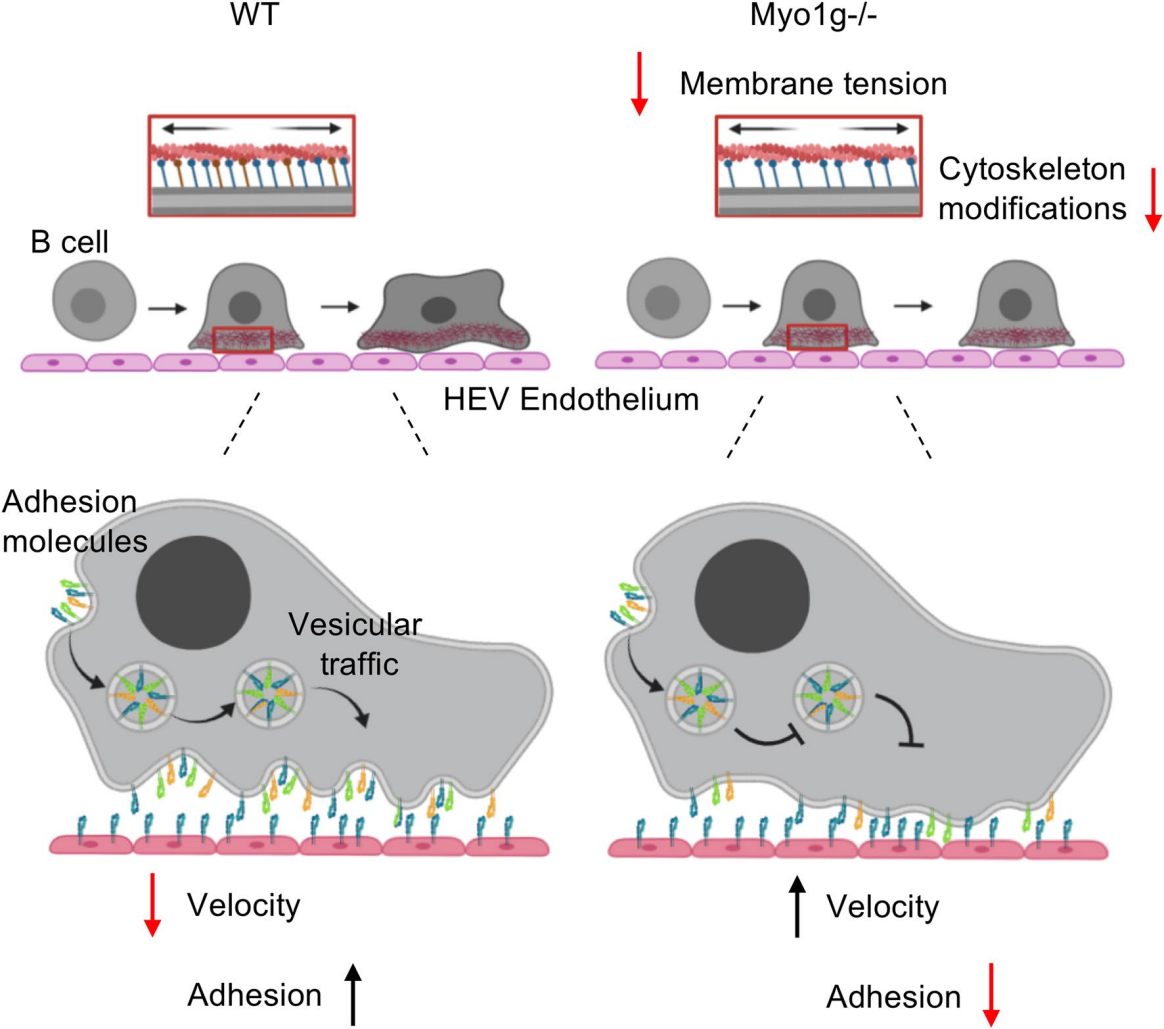
The absence of Myo1g limits the ability of B lymphocytes to generate morphological changes during migration. Schematic illustration of the role of Myo1g during activated B lymphocytes migration. In absence of Myo1g activated B lymphocytes have a lower capacity to exert changes in their morphology and lower surface expression of adhesion molecules. These changes increase their speed of movement, reducing the interaction between the B lymphocyte and the endothelium, directly affecting homing of B lymphocytes towards the inguinal lymph node. Created with BioRender.com.

In this work, we used an in vivo model to analyze the participation of Myo1g during the migration of activated B lymphocytes. We observed that in the absence of Myo1g, LPS + IL-4 activated B lymphocytes had reduced capacity to interact with the endothelium of postcapillary venules II, III, and IV in inguinal LNs. The deficiency was manifested by increased rolling velocities and reduced adhesion and transmigration. In the absence of Myo1g, activated B lymphocytes reached fewer lymph nodes, indicating this myosin's relevance in B cells' homing^20, 21^. We reported recently that another class 1 myosin (Myo1e) regulates adhesion and migration of B lymphocytes by mobilizing FAK and allowing the activation of the FAK-PI3K-Rac1 pathway^32^. Myo1e has an SH3 domain that allows for protein–protein interactions^13, 16, 30, 32^. However, Myo1g lacks an SH3 domain. Therefore, the mechanism by which Myo1e and Myo1g participate in the migration of B lymphocytes should be different. Myo1c, another short tail class I myosin present in B lymphocytes' microvilli, takes part in B lymphocytes' ability to modify their cytoskeleton^33^. These results suggest that although short-tailed class I myosins (such as Myo1c and Myo1g) lack an SH3 domain, they are relevant for the actin cytoskeleton's plasticity in B lymphocytes by a still poorly defined mechanism. We used microfluidic devices to characterize further activated Myo1g^−/−^ B lymphocytes; these devices allowed us to analyze 3D migration in vitro^26, 27^. The results showed that activated Myo1g-deficient B lymphocytes moved faster within a 10 μm channel. Previous work has demonstrated that in the absence of Myo1g, T cells adhere less efficiently to ICAM-1 and migrate faster than WT T cells^27^, suggesting that the absence of Myo1g reduces the adhesive abilities of T and B lymphocytes. In 3D migration models, round morphology favors displacement, so activated Myo1g^−/−^ B lymphocytes may move more easily. Another possibility in the 3D model is that activated Myo1g^−/−^ B lymphocytes have reduced expression of VLA-4, a Fibronectin ligand, the substrate that covers our device. Then, the interaction could be weak allowing an increased speed in migration. However, VLA-4 is not the only Fibronectin' receptor present on B lymphocytes, so other receptors should be evaluated to rule out their participation^34^. Similarly, the absence of Myo1g decreases the adhesion of B lymphocytes to other substrates^20, 21^. Myo1g also regulates the recycling of lipid raft-associated receptors^21^. Myo1g has a PH-like domain that allows it to interact with lipid rafts^19, 22^, and several adhesion molecules such as selectins, integrins are present in these lipid domains. Therefore, problems with mobilization or recycling could be associated with lower adhesion molecules' expression in activated Myo1g^−/−^ B lymphocytes. However, it remains to be investigated whether Myo1g can also regulate the signaling pathways promoting these adhesion molecules' de novo expression. It has been observed that Myo1g regulates actin cytoskeletal dynamics and the elasticity of the plasma membrane. These factors favor the generation of lamellipodia and filopodia, which are critically involved in these processes^11, 29^. Our results showed that Myo1g is concentrated at the poles of the cells when adopting a migratory phenotype. During cell migration, the changes generated in the cytoskeleton require a force to be exerted on the membrane. This force is provided by actin filament dynamics, promoting cell shape modifications and forming membrane structures such as filopodia and lamellipodia^10, 12, 35^. These mechanical forces exerted on the membrane are regulated by the membrane's tension regulated by various proteins, including Myo1g^14, 29, 36^. In previous work, we reported that in the absence of Myo1g, LPS + IL-4 activated B lymphocytes had decreased membrane tension^17^. In addition to regulating elasticity, membrane tension must control the strength between the cytoskeleton and adhesion molecules through a clutch that joins the actin filaments with the integrins^10, 37, 38^. Moreover, it has been reported that membrane tension acts through the PLD2–mTORC2 pathway to inhibit actin nucleation via the WAVE2 complex. These molecules could also participate in the signaling that favors rearrangements in the cytoskeleton^39–41^. Therefore, we propose that when membrane tension of activated B lymphocytes is reduced (by the absence of Myo1g), the cells have less capacity to produce stable membrane structures to convert them into a migratory phenotype. In conclusion, here we present pieces of evidence about the role of Myo1g in regulating the adhesion of B lymphocytes to HEV, affecting the formation of membrane projections necessary for a migratory phenotype. Thus, the absence of Myo1g decreases B lymphocytes' interaction with the endothelium, which, together with the lower expression of adhesion molecules, reduces activated B lymphocytes' capacity to enter inguinal LNs. Additionally, in this work, we show evidence for the role of Myo1g in B lymphocyte migration in vitro and in vivo. Myo1g regulates activated B lymphocytes' adhesion to the endothelium of HEV and membrane tension that affects the cell membrane's elasticity, the formation of membrane projections, and the acquisition of a migratory phenotype. Finally, our findings give information about how Myo1g regulates cytoskeleton rearrangements during the B lymphocyte CXCL13-dependent migration process. Though our in vivo model was focussed on LN homing, lymphocyte migration is a conserved process. Hence, the phenomenon triggered by the absence of Myo1g could be present when Myo1g^−/−^ B lymphocytes migrates toward other sites, for example, Peyer' Patches, where homing is CXCL13-dependent^42^. It will be interesting to analyze the functional effect of the activated Myo1g^−/−^ B lymphocytes' irregular migration. In Fig. 6, a schematic illustration is shown that combines the results of this work with previous reports about the function of Myo1g in murine B lymphocytes. In this way, a model is proposed in which Myo1g could regulate different functions during the migration of B lymphocytes to the inguinal lymph node.

## Methods

### Mice

8–10 weeks old female wild type or Myo1g^−/−^ C57BL/6 J mice were used in all experiments. The mice were bred and maintained at the animal facility of Cinvestav-IPN (Mexico City, Mexico). The Animal Care and Use Committee of Cinvestav-IPN approved all experiments. All methods were performed in accordance with the relevant guidelines and regulations. In vivo methods were carried out in compliance with the ARRIVE guidelines.

### Lymphocyte isolation and flow cytometry

Splenic mononuclear cells were isolated by Ficoll-paque Plus (GE Healthcare, Little Chalfont, UK) density gradient separation, and B220 + cells were enriched by panning, using plastic dishes coated with α-Thy-1 monoclonal antibody ascites (NIM-R1) (Chayen and Parkhouse, 1982). For B-cell activation, 2 × 10^6^ cells were incubated 48 h at 37 °C, and 5% CO_2_ in 1 ml RPMI 1640 (Life Technologies, Grand Island, NY) supplemented with 10% fetal bovine serum (Thermo Fisher Scientific, Waltham, MA). Stimulation was induced by 48 h incubation with LPS from Escherichia coli O55: B5 (20 μg/ml) (Sigma-Aldrich, St Louis, MO) plus the addition of IL-4 (10 U/ml) (R&D Systems, Minneapolis, MN). This methodology was carried out following a previously published protocol^32^. For immunostaining, the Fc receptors were blocked using 10% goat serum. Cell suspensions were washed with PBS containing 1% bovine serum albumin (BSA) (Thermo Fisher Scientific) and 0.01% NaN_3_ (PBA). One million cells were stained 15 min using the antibodies described in the following section. After incubation, the cells were washed with PBA and fixed with 1% formaldehyde in PBS (0.5% albumin, 0.01%NaN3, 100 ml PBS). Doublets were excluded by gating on FSC-H versus FSC-A, and the lymphocytes were identified by their scatter properties (FSC-A versus SSC-A). Compensation was performed using single-stained cells for each of the fluorochromes used. The cells were evaluated using a BD LSR Fortessa flow cytometer (Becton–Dickinson, San Jose, CA) and analyzed using FlowJo v.10 software (Tree Star, Ashland, OR). All experiments were performed according to proper flow cytometry guidelines (Cossarizza et al., 2017). This methodology was carried out following a previously published protocol^32^.

### Antibodies and reagents

The antibodies used were: polyclonal IgG anti Myo1g, anti-B220/CD45R (clone RA3-6B2, BioLegend), anti-CD29 (clone hm *B*1-1, BioLegend), anti-LFA-1 (clone Hl111, BioLegend), anti-CD62L (clone DREG-56, BioLegend), anti-CD44 (clone IM7, BioLegend), anti-CXCR5 (clone 2G8, BD Pharmigen), anti-CCR7 (Clone 4B12, ThermoFisher) anti CXCR4 (Clone 2B11, Invitrogen). TRITC-Phalloidin (Thermo Fischer, Scientific), Hoechst 33342 (Thermo Fischer, Scientific), murine CXCL12 and CXCL13 (PeproTech).

### Intravital microscopy

WT host mice were anesthetized by intraperitoneal injection of xylazine (12.5 mg/kg) and ketamine hydrochloride (125 mg/kg) (Sanofi, Mexico-City, Mexico). The inguinal lymph node was then inoculated with 100 μl CXCL13 (25 ng/ml) (PeproTech) to promote cell recruitment. One hour later, 1 × 10^7^ Hoechst 33342-labeled, LPS + IL-4 activated B cells were injected via the cannulated carotid artery. Venules of the inguinal lymph node were recorded using an intravital upright microscope (Axioscope, Model A1, Zeiss, Jena, Germany) with a 40 × and 0.75 saline immersion objective (Zeiss). Videos and images were analyzed using ImageJ (NIH, Bethesda, MD. USA) and Zen Blue Edition 2.5 software (Zeiss, Microscopy). The venules' diameter, the number of adherent cells, the number of transmigrated cells, and the cells' velocity were measured using ImageJ. Cell flux, blood flow, and rolling velocity were analyzed by Zen Blue Edition 2.5 software (Zeiss, Microscopy). This methodology was carried out following a previously published protocol^32^.

### Microfluidic device fabrication and imaging

Microfluidic devices were fabricated by standard soft lithography techniques that have been described before^43^. Devices are made with polydimethylsiloxane (PDMS, Sylgard 184, Dow Corning). The microfluidic device comprises two parallel chambers separated by 150 μm, each with a height of 15 μm, a width of 500 μm, and a length of 5 mm. A series of perpendicular microchannels connect both chambers with alternated widths of 5, 10, and 15 μm, and the same height as chambers allows cell migration from one chamber to the other. Independent inlets and outlets are punched for each chamber, and media reservoirs are bonded to them to maintain cells viably and exchange solutions inside the device. Microfluidic devices were treated with Fibronectin before cell seeding to improve cell adhesion. LPS + IL-4 activated B lymphocytes were seeded into microfluidic chambers by flowing a cell suspension (10^5^ cells mL^−1^) into one of the chambers, waiting 10 min for cells to adhere and then washing excess cells with fresh media. Subsequently, we applied CXCL13 or CXCL12 (PeproTech, Rocky Hill, NJ, USA) on the chamber's opposite side. B cell movement inside the microchannels was imaged for 30 min at intervals of 20 s in a confocal microscope. A minimum of 25 cells per chamber, per treatment condition, was analyzed.

### Migration-phenotype assay

A Zigmond chamber (Neuroprobe, Gaithersburg, MD) was used to quantify the migratory phenotype. Briefly, 1 × 10^6^ LPS + IL-4 activated B lymphocytes from WT and Myo1g^−/−^ were suspended in 0.5 ml RPMI 1640 supplemented with 10% fetal bovine serum immediately plated onto glass coverslips. The glass coverslips were previously coated with Fibronectin (2.5 μg/ml) (Sigma-Aldrich) or Poly-l-Lysine (Sigma-Aldrich), and the cells were incubated 30 min at 37 °C and 5% CO_2_ to allow attachment. The coverslips, with the cells attached, were gently washed with PBS. One of the grooves in the Zigmond chamber was filled with supplemented medium (∼70 μl), and the other with CXCL13 (2.5 μg/μl) dissolved in the supplemented medium. Subsequently, cells were incubated for 1 h at 37 °C and 5% CO_2_ to allow sensing of the CXCL13 gradient. Migration tracks of at least 50 activated lymphocytes of WT and Myo1g^−/−^ mice, in three independent experiments, were analyzed using ImageJ software (National Institutes of Health, Bethesda, MD). This methodology was carried out following a previously published protocol^21^.

### Membrane-projections assay

1 × 10^6^ LPS + IL-4 activated B lymphocytes from WT and Myo1g^−/−^ mice were suspended in 0.5 ml RPMI 1640 supplemented with 10% fetal bovine serum immediately plated onto glass coverslips coated with Fibronectin (2.5 μg/ml) (Sigma-Aldrich). Cells were incubated one h at 37 °C and 5% CO_2_ to allow adhesion. Subsequently, cells were fixed and stained with TRITC-Phalloidin for 20 min. At least 50 activated B lymphocytes of WT and Myo1g^−/−^ mice, in three independent experiments, were analyzed using ImageJ software (National Institutes of Health, Bethesda, MD). This methodology was carried out following a previously published protocol^21^.

### Trans-well assay

1.5 × 10^4^ LPS + IL-4 activated WT and Myo1g^−/−^ B lymphocytes were placed in the upper compartment of 5 μm pore-trans-well chamber (Corning) in RPMI 1640 (Life Technologies) without Fetal Calf Serum (FCS). Ten minutes before seeding the cell suspension in the top compartment, we added FCS or CXCL12 (100 ng/ml) to the lower well. The assembled chamber was incubated 4 h at 37 °C and 5% CO_2_. After incubation, the cells in the lower compartment were counted, and we calculated the percentage of migrating cells from the number of initial cells seeded in the top chamber.

### Statistical analysis

Data are presented as the arithmetic mean with standard deviations; Student’s t-test was used for evaluating statistical differences. A p-value of < 0.05 was considered statically significant. The p values are represented as *p < 0.05, **p < 0.01, ***p < 0.001, and ****p < 0.0001, and the number of samples or cells (n) used are mentioned in each figure legend. This analysis was carried out following a previously published protocol^21^.

## Supplementary Information

Supplementary Information 1.

Supplementary Video 1.

Supplementary Video 2.

Supplementary Video 3.

Supplementary Video 4.

## Data Availability

Raw data used and analyzed during the current study are available from the corresponding author on reasonable request.
