# Supplementary material for: Myo1g is required for efficient adhesion and migration of activated B lymphocytes to inguinal lymph nodes
Source: Sci Rep. 2021 Mar 30;11:7197. doi: 10.1038/s41598-021-85477-y (PMC8009870; doi:10.1038/s41598-021-85477-y)
Supplement: Supplementary file 1 — Supplementary Information 1. [file 41598_2021_85477_MOESM1_ESM.pdf]

Supplementary Information

Myo1g is required for efficient adhesion and migration of activated B lymphocytes to inguinal lymph nodes

Cruz-Zárate D[1, 3*], López-Ortega O[1*], Girón-Pérez D[1*], Gonzalez-Suarez AM[2], García-Cordero JL[2], Schnoor M[1], *Santos-Argumedo L[1]

These authors equally contributed to this manuscript

[1]Departamento de Biomedicina Molecular, Centro de Investigación y de Estudios Avanzados del Instituto Politécnico Nacional (CINVESTAV-IPN), México City

[2]Unidad Monterrey, Centro de Investigación y de Estudios Avanzados del Instituto Politécnico Nacional (CINVESTAV-IPN), Monterrey NL, México

[3]Departmento & Posgrado en Inmunologia, Escuela Nacional de Ciencias Biologicas del Instituto Politécnico Nacional (ENCB-IPN), México City, México

Corresponding author information

Leopoldo Santos-Argumedo

Av. Instituto Politécnico Nacional 2508,

San Pedro Zacatenco,

07360 Ciudad de México

Telephone number: +52 (55) 5747-3800 ext: 5020

Fax number: +52 (55) 5747-3800 ext: 5020

E-mail Address: lesantos@cinvestav.mx

Supplementary Figure S1

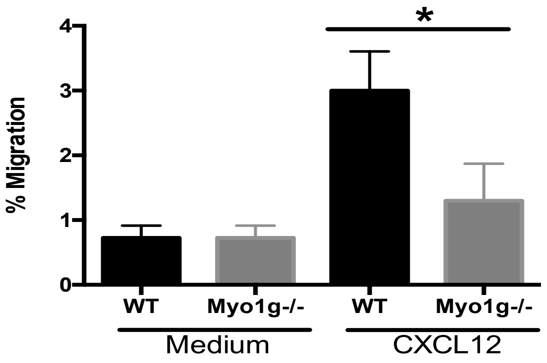

Trans-well migration of LPS + IL-4 activated WT and Myo1g-/- B lymphocytes.

$1.5x10^4$ LPS + IL-4 activated WT, and Myo1g-/- B lymphocytes were placed in the 5μm pore-trans-well chamber's upper compartment. FCS or CXCL12 (100ng/ml) was added to the lower chamber. Data show the percentage of migration of activated B lymphocytes after 4h at 37°C. ****P<0.0001.

Supplementary Video S2. Representative video of LPS + IL-4 activated WT B lymphocytes moving through the venules of an inguinal lymph node previously (1 h) inoculated with CXCL13 (25ng/ml. (40x objective)

Supplementary Video S3. Representative video of LPS + IL-4 activated Myo1g-/- B lymphocytes moving through the venules of an inguinal lymph node previously (1 h) inoculated with CXCL13 (25ng/ml. (40x objective).

Supplementary Figure S4

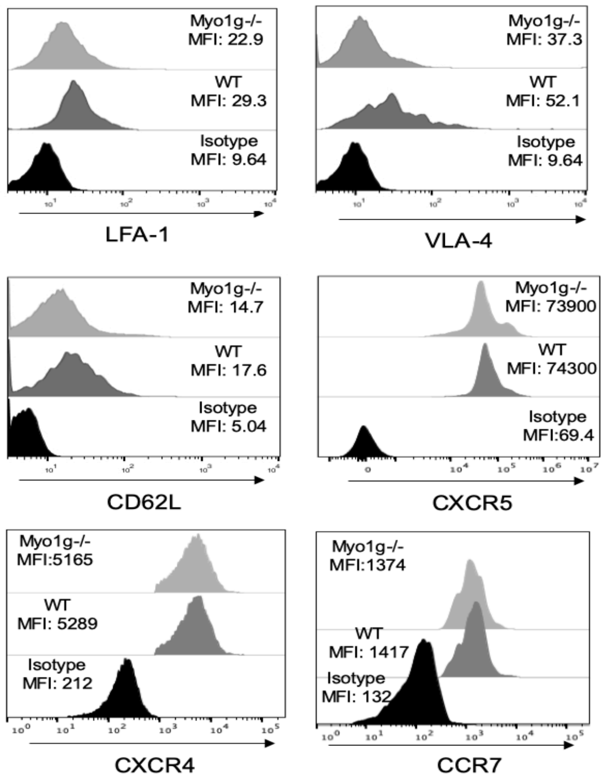

Surface expression of adhesion molecules, integrins, and chemokine receptors in LPS + IL-4 activated WT and Myo1g-/- B lymphocytes. Representative histograms and mean fluorescence intensity values of surface expression of adhesion molecules, integrins and chemokine receptors and isotype control antibodies in LPS + IL-4 activated WT and Myo1g-/- B lymphocytes.

Supplementary Figure S5

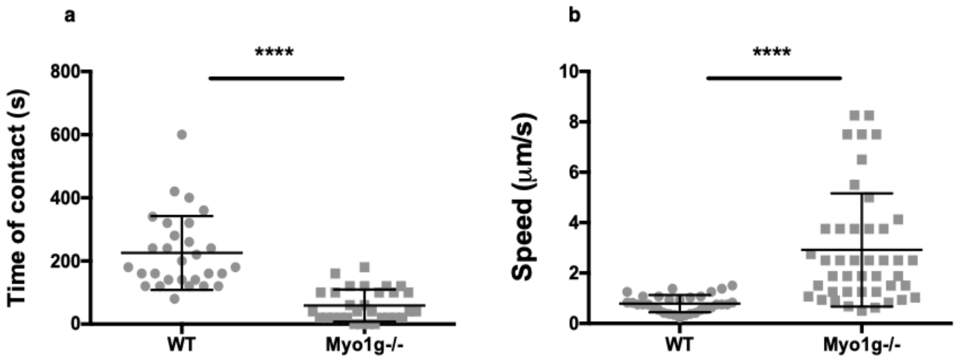

Motility of LPS + IL-4 activated WT and Myo1g-/- B lymphocytes in a confined 3D microchannel with CXCL12 as a chemoattractant. (a) Time of contact (s) of LPS + IL-4 activated WT or Myo1g-/- B lymphocytes within 10 μm microchannel wall stimulated by a gradient of CXCL12. (b) speed (μm/s) of B lymphocytes moving through a 10 μm microchannel. Data are presented as mean ± s.d. *P<0.01.

Supplementary Video S6. Representative video of LPS + IL-4 activated WT B lymphocytes moving through 10μm microchannel under CXCL13 gradient. (10x objective).

Supplementary Video S7. Representative video of LPS + IL-4 activated Myo1g-/- B lymphocytes moving through 10μm microchannel under CXCL13 gradient. (10x objective).

Supplementary Figure S8

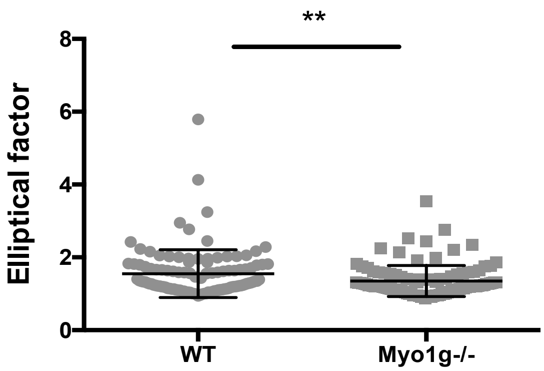

Elliptical factor in LPS + IL-4 activated B lymphocytes. Measurement of the elliptical factor of LPS + IL-4 activated WT or Myo1g-/- B lymphocytes on fibronectin-coated slides. Data correspond to three independent experiments. Data are presented as mean ± s.d. ***$P<0.0001$.*
